# Alternatively Activated Macrophages Are Host Cells for *Chlamydia trachomatis* and Reverse Anti-chlamydial Classically Activated Macrophages

**DOI:** 10.3389/fmicb.2019.00919

**Published:** 2019-05-07

**Authors:** Illya Tietzel, Alison J. Quayle, Rey A. Carabeo

**Affiliations:** ^1^Department of Natural Sciences, Southern University at New Orleans, New Orleans, LA, United States; ^2^Department of Microbiology, Immunology, and Parasitology, Louisiana State University Health Sciences Center, New Orleans, LA, United States; ^3^School of Molecular Biosciences, Washington State University, Pullman, WA, United States

**Keywords:** Chlamydia, macrophages, pathogenesis, alternative activation of macrophages, immunomodulation, GBP, indoleamine 2, 3-dioxygenase

## Abstract

The obligate intracellular pathogen *Chlamydia trachomatis* (Ctr) is the causative agent of the most common form of sexually transmitted disease in the United States. Genital infections with *C. trachomatis* can lead to inflammatory tissue damage followed by scarring and tissue remodeling during wound healing. Extensive scarring can lead to ectopic pregnancy or infertility. Classically activated macrophages (CA mϕ), with their anti-microbial effector mechanisms, are known to be involved in acute inflammatory processes during the course of infection. In contrast, alternatively activated macrophages (AA mϕ) contribute to tissue repair at sites of wound healing, and have reduced bactericidal functions. They are present during infection, and thus potentially can provide a growth niche for *C. trachomatis* during a course of infection. To address this question, macrophages derived from CD14-positive monocytes magnetically isolated from peripheral blood mononuclear cells (PBMC) were treated with interferon-γ or interleukin-4 to produce CA mϕ or AA mϕ, respectively. Confocal microscopy of chlamydial inclusions and quantification of infectious yields revealed better pathogen growth and development in AA mϕ than CA mϕ, which correlated with the reduced expression of indoleamine 2,3-dioxygenase, a known anti-chlamydial effector of the host. Furthermore, AA mϕ stained strongly for transferrin receptor and secreted higher amounts of anti-inflammatory interleukin-10 compared to CA mϕ, characteristics that indicate its suitability as host to *C. trachomatis*. CA, AA, and resting mϕ were infected with Ctr serovar L2. The data suggest that IL-10 produced by infected AA mϕ attenuated the anti-chlamydial function of CA mϕ with growth recovery observed in infected CA mϕ in the presence of infected, but not mock-infected AA mϕ. This could be related to our observation that IL-10 treatment of infected CA mϕ promoted better chlamydial growth. Thus, in addition to serving as an additional niche, AA mϕ might also serve as a means to modulate the immediate environment by attenuating the anti-chlamydial functions of nearby CA mϕ in a manner that could involve IL-10 produced by infected AA mϕ.

## Introduction

*Chlamydia trachomatis* (Ctr) is an obligate intracellular bacterium and is the bacterial pathogen that causes the most common sexually transmitted diseases in the United States. In 2016 over 1.5 million cases of *C. trachomatis* infections were reported for the United States (Centers for Disease Control and Prevention, 2017). Complications of chlamydial infections involve asymptomatic infections that are spread unknowingly, pelvic inflammatory disease, extensive scarring, infertility, atopic pregnancies, or adverse pregnancy outcomes (Haggerty et al., 2010; Centers for Disease Control and Prevention, 2017). The prevalence of *C. trachomatis* infections and costs resulting from the complications of these infections render it a major concern of public health.

*Chlamydia trachomatis* can infect epithelial cells that line the genital tract. Monocytes and macrophages are also found in these tissue sites, and thus likely encountered by *C. trachomatis* (Pudney et al., 2005). The outcome of this interaction in the context of genital infections remains to be elucidated. A study found that monocytes do not allow *Chlamydia pneumoniae* to complete its intracellular developmental cycle but may do so transiently in macrophages that were characterized of a type that was CD206-positive (Wolf et al., 2005).

There are different types of macrophages defined by their activation mode, phenotype, and biological function. One of the earliest reports of so called alternatively activated macrophages described the cell surface expression of high levels of the mannose receptor and the reduction in levels of secreted pro-inflammatory cytokines by murine macrophages treated with the cytokine interleukin-4 (IL-4) (Stein et al., 1992). Since the initial report, additional phenotypes of IL-4-treated macrophages have been discovered that distinguish this population from the interferon-γ-activated macrophages (Gordon, 2003; Davies et al., 2013). For example, classically activated macrophages are activated by interferon-γ (IFN-γ), they express high levels of CD64, anti-microbial effectors like inducible nitric oxide synthase (iNOS) or indoleamine-2,3-dioxygenase (IDO), cytokines like interleukin (IL)-12p70, and are found at sites of inflammation (Gordon, 2003). Alternatively activated macrophages are activated by IL-4, IL-13 or other cytokines, express high levels of CD206 (mannose receptor), anti-inflammatory cytokines like IL-10 or TGF-beta, and are associated with sites undergoing tissue repair (Gordon, 2003). In addition, their anti-microbial functions are attenuated (Kahnert et al., 2006). Alternatively activated macrophages are often found at sites of tissue damage, including damage arising from infection. Vicetti Miguel et al. (2013) reported that endometrial Chlamydia infection promoted alternative activation of macrophages and Wolf et al. (2005) found mature chlamydial inclusions in CD206-positive macrophages, which led us to investigate the nature of the interaction of *C. trachomatis* with this cell type. We determined that alternatively activated macrophages are suitable hosts for *C. trachomatis*, with the pathogen successfully completing its developmental cycle to generate infectious particles. Furthermore, *C. trachomatis* stimulated the production of IL-10, which may be related to the observed attenuated anti-microbial functions of classically activated macrophages in co-culture conditions. Collectively, these results point to Chlamydia taking advantage of the reduced anti-microbial function of alternatively activated macrophages to promote its growth, while simultaneously manipulating the immediate environment through enhanced IL-10 production by the alternatively activated macrophage host.

## Results

### *Chlamydia trachomatis* Replicates in AA mϕ, but Not CA mϕ

**FIGURE 1:**
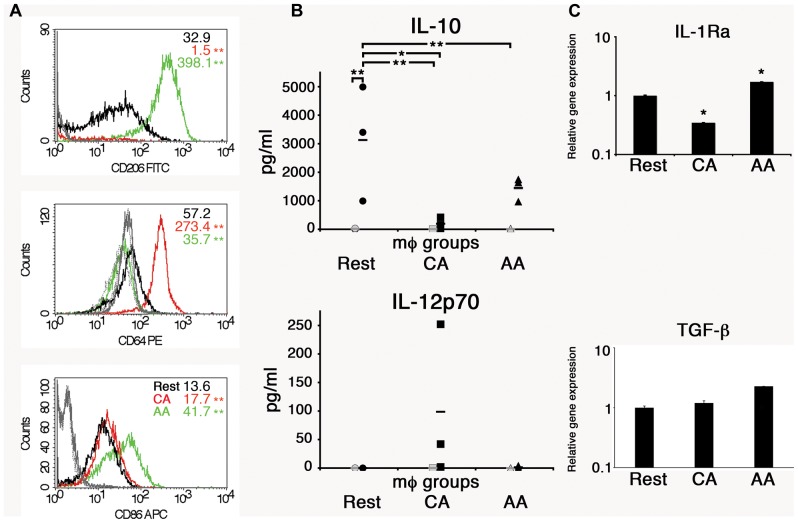
Anti-inflammatory properties of AA mϕ. **(A)** FACS analysis showed high levels of CD206 and low levels of CD64 for AA mϕ (green, isotype solid gray), CA mϕ (red, isotype dashed gray) displayed opposite pattern. Resting mϕ (black, isotype dotted-dashed gray) showed intermediate phenotype. The overall *p*-value for all surface markers was less than 0.0001. ^∗∗^*p*-value ≤ 0.01 compared to resting macrophages. **(B)** Production of anti-inflammatory IL-10 and inflammatory IL-12p70 in cell culture supernatants of alternatively activated mϕ (triangles), classically activated mϕ (squares), and of resting mϕ (circles) left untreated (gray symbols) or stimulated for 24 h with LPS (black symbols) compiled from three independent experiments. Means are indicated by horizontal bars. Overall *p*-value for IL-10 was 0.0032. ^∗∗^*p* ≤ 0.01; ^∗^*p* ≤ 0.05 for individual group comparisons. The overall *p*-value for IL-12p70 was 0.2308 and deemed not significant. Posttests were not performed because the *p*-value was greater than 0.05. AA mϕ responded with higher amounts of IL-10 and lower amounts of IL-12p70 when compared with CA mϕ. **(C)** The gene expression of anti-inflammatory markers such as IL-1Ra and TGF-beta differs between AA and CA mϕ. Real time PCR data were statistically evaluated with group-wise comparison and Relative Expression Software Tool (Pfaffl et al., 2002). ^∗^*p* ≤ 0.05 compared to resting mϕ as control group. Mean values of triplicates with standard deviations are depicted.

CD14+ monocytes were converted to macrophages by incubation in macrophage-colony stimulating factor (M-CSF) for 5 days. Non-adherent cells were discarded, and the remaining adherent macrophages (mϕ) were activated with either IL-4 or IFN-γ to alternatively activated (AA) or classically activated (CA) macrophages. To verify the activation states, cells were analyzed for cell surface markers, including mannose receptor (CD206), high-affinity Fc gamma receptor (CD64), and a co-stimulatory molecule CD86. The mean fluorescence intensity (MFI) of CD206 was 32.9 for resting macrophages, 1.5 for CA mϕ, and 398.1 for AA mϕ (Figure 1). The MFI of CD64 was 57.2 for resting macrophages, 273.4 for CA mϕ, and 35.7 for AA mϕ. The MFI of CD86 was 13.6 for resting macrophages, compared to 17.7 for CA mϕ and 41.7 for AA mϕ. Macrophages activated with IL-4 expressed high levels of CD206, consistent phenotypically with the AA mϕ, while those activated with IFN-γ displayed high cell surface expression of CD64 (Figure 1). The IL-4 activated macrophages also displayed higher levels of cell surface CD86 relative to the IFN-γ-activated population. The non-activated group (resting mϕ) displayed low levels of CD206 and CD86 relative to the putative AA mϕ subset. Overall, IL-4 activation of macrophages generated CD206^hi^ CD64^lo^ CD86^hi^ mϕ consistent with alternative activation, while IFN-γ treatment yielded a population displaying CD206^-^ CD64^hi^ CD86^lo^ phenotype, suggestive of classical activation. Resting mϕ had a CD206^lo^ CD64^lo^ CD86^lo^ phenotype.

The macrophage types were also analyzed for other anti-inflammatory markers such as interleukin-1 receptor antagonist (IL-1 Ra) and transforming growth factor-beta (TGF-β). Unstimulated AA mϕ showed high mRNA levels of IL-1 Ra (1.7-fold increase), but CA mϕ did not (0.3-fold expression of resting macrophages). The relative expression of TGF-β increased for AA mϕ 2.3-fold. Relative expression for resting macrophages was 1 with a standard deviation of 0.068, for CA mϕ 1.197 with a standard deviation of 0.107, and for AA mϕ 2.275 with a standard deviation of 0.003.

**FIGURE 2:**
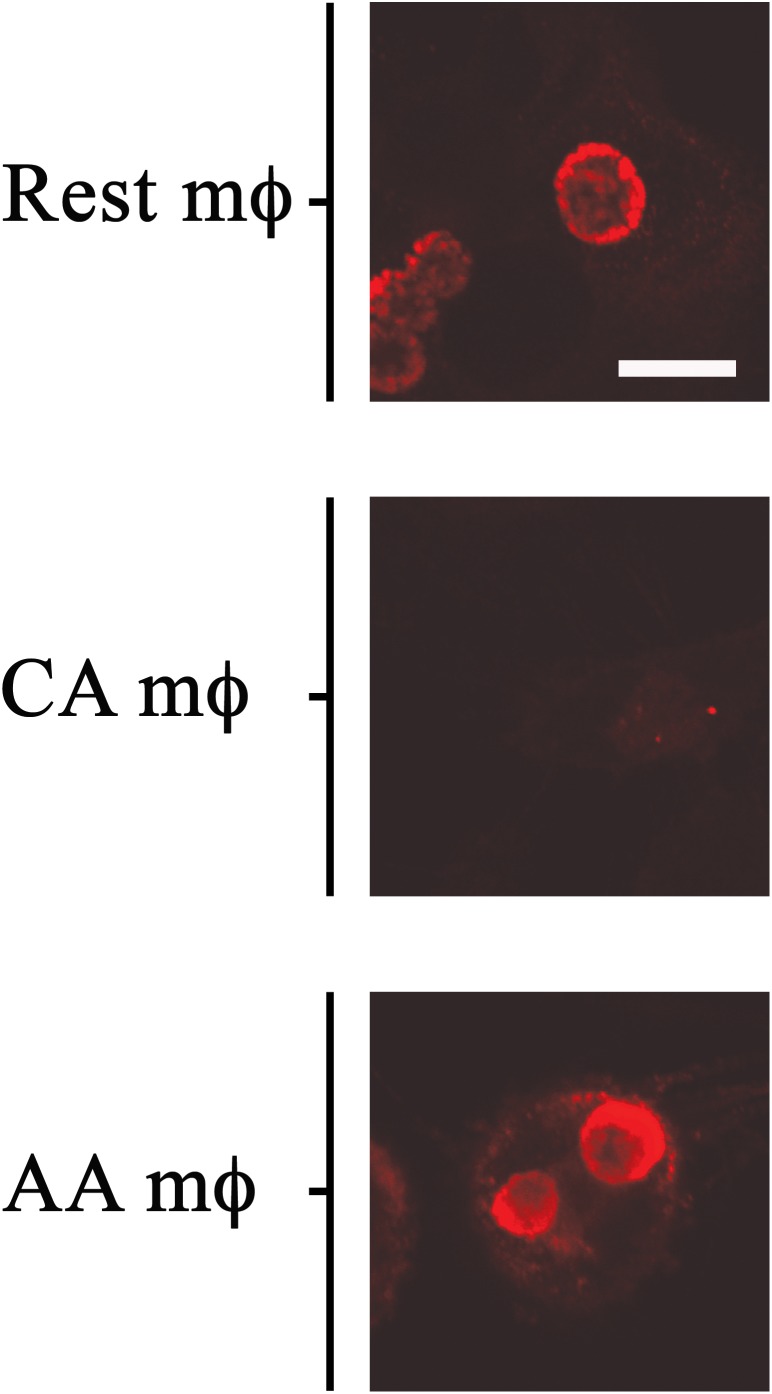
Growth of chlamydial inclusions in AA mϕ, but not in CA mϕ. Confocal micrographs of immunofluorescence analysis of *Chlamydia trachomatis* of human resting (Rest), classically activated (CA) or alternatively activated (AA) macrophages about one day post-infection. White scale bar represents 10 μm and data are representative of at least three independent experiments.

The *in vitro*-activated AA and CA mϕ were evaluated for their ability to support growth of *C. trachomatis*. Resting mϕ were included for comparison. Macrophages seeded on glass coverslips were infected with *C. trachomatis* at a multiplicity of infection (MOI) of one. Infection was allowed to proceed for 20–24 h, and samples were either fixed for indirect immunofluorescence confocal microscopy to evaluate inclusion size, or lysed to collect infectious particles to evaluate the extent of chlamydial development. Regeneration of infectious elementary bodies (EBs) would indicate completion of the biphasic developmental cycle. Images of inclusions shown in Figure 2 are representative of 200 cells (91 resting mϕ, 41 CA mϕ, 68 AA mϕ) from five independent trials. Larger inclusions in resting and AA mϕ were readily observed in comparison to those found in CA mϕ. Because inclusion size generally correlates with the level of *C. trachomatis* replication, the results suggested that resting and AA mϕ were better able to support chlamydial growth, with observations for the latter characteristic of an attenuated anti-microbial function. *C. trachomatis* inclusions in CA mϕ were generally smaller in size, which was expected considering that IFN-γ-stimulated macrophages are known to eliminate efficiently intracellular infections.

**FIGURE 3:**
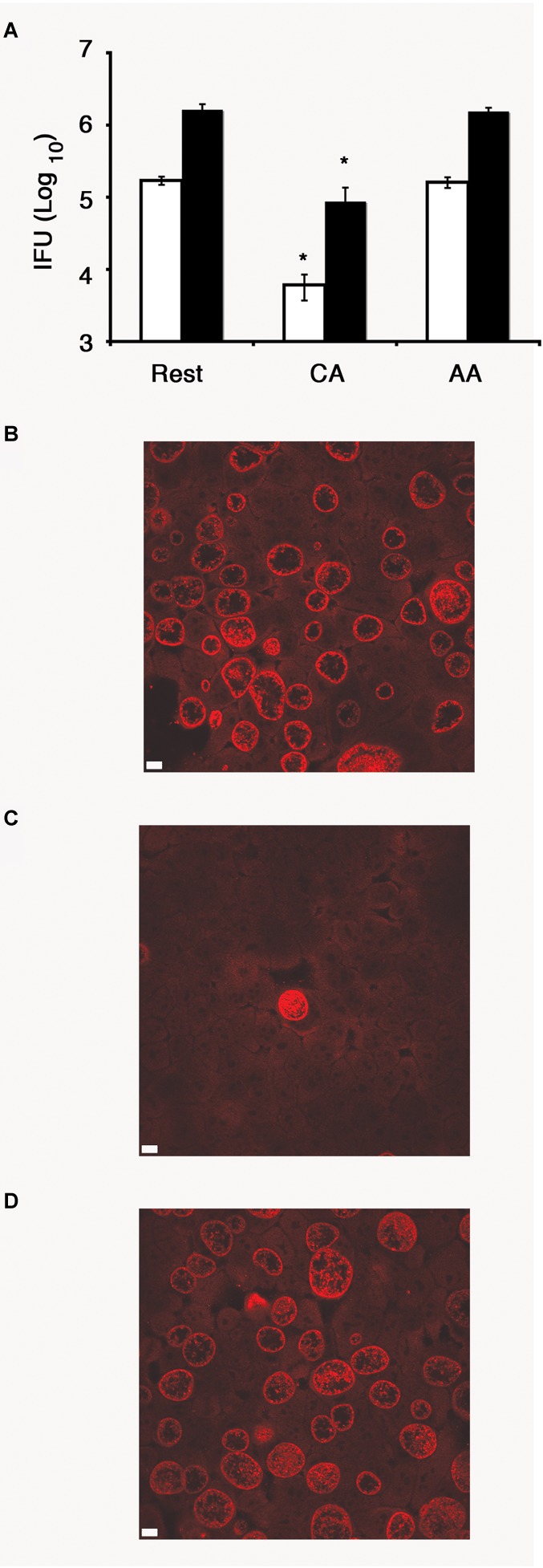
Recovery of inclusion forming unit (IFU) reduced in CA mϕ, but not in AA mϕ. **(A)** Quantitative analysis of recovery of IFU from lysates of resting (Rest), classically activated (CA) or alternatively activated mϕ (AA) that were originally inoculated with a MOI of one (white bars) or five (black bars), respectively. IFU numbers for CA mϕ are dramatically reduced in comparison to resting mϕ with a value of *p* ≤ 0.01 (^∗^) indicating statistical significance. No statistically significant differences were found for AA mϕ when compared to resting mϕ. Confocal micrograph of HeLa cells with immunofluorescently labeled IFUs recovered from resting **(B)**, classically activated **(C)** or alternatively activated mϕ **(D)** that were inoculated at an MOI of one. The white scale bar indicates 10 μm.

To determine whether *C. trachomatis* can complete its developmental cycle in the AA mϕ, we recovered EBs at 1 day post-infection and used them to infect HeLa cells for 24 h. A significantly higher IFU count was detected in AA mϕ compared to CA mϕ indicating that a markedly higher amount of chlamydial bodies completed their developmental cycle in the former subpopulation (Figure 3). *Chlamydia trachomatis* was also able to complete its developmental cycle in resting macrophages.

### Effects of Infection on the Expression ofCell Surface Markers and SelectHallmark Genes

**FIGURE 4:**
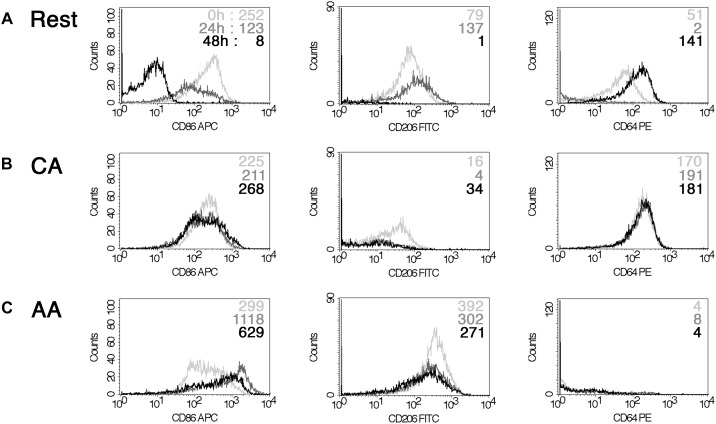
Impact of chlamydial infection on mϕ phenotype. FACS analysis of a time-course of infection with *Chlamydia trachomatis* (0, 24, and 48 h) indicated down-regulation of CD86 for resting mϕ **(A)**. However, CA mϕ **(B)** and AA mϕ **(C)** maintained their CD64 high and CD206 levels relatively to each other. 0 h time-points are represented with light gray graphs and upper mean fluorescence intensity (MFI) values of each histogram. 24 h are indicated in dark gray or middle MFI values, 48 h appear black or as bottom MFI results.

Infection did not significantly alter the cell surface marker phenotype. Cell surface expression of CD206, CD86, and CD64 was monitored by flow cytometry in mock- and *C. trachomatis*-infected cells at 0, 24, and 48 h post-infection (Figure 4). In mock-infected resting macrophages, the MFI values of CD86 were 252, and 123 or 8 for the different time points post-infection. Values for CD206 were 79, 137, and 1. Data for CD64 were 51, 2, and 141. In mock-infected AA mϕ, the MFI values of CD86 were 299, and 1118 or 629 for the different time points post-infection. Values for CD206 were 392, 302, and 271. Data for CD64 were 4, 8, and 4. In mock-infected CA mϕ, the MFI values of CD86 were 225, and 211 or 268 for the different time points post-infection. Values for CD206 were 16, 4, and 34. Data for CD64 were 170, 191, and 181. Chlamydia-infected AA mϕ maintained their high level of CD206, with no significant changes to CD206 levels across all time points. Expression of CD86 in infected AA mϕ stayed at high level or even increased further starting at 24 h post-infection (p.i.). CD86 and CD64 expression remained steady in infected CA mϕ, with very little change across all time points post-infection. Interestingly, chlamydial infection down-regulated expression of CD86 in resting mϕ starting at 24 h p.i. A similar downregulation, albeit to a smaller extent was observed for CD206 at 48 h p.i. relative to earlier time points. CD64 for CA mϕ remained on a relatively high level compared to the other macrophage types. Resting mϕ showed a slight increase in CD64 cell surface expression.

**FIGURE 5:**
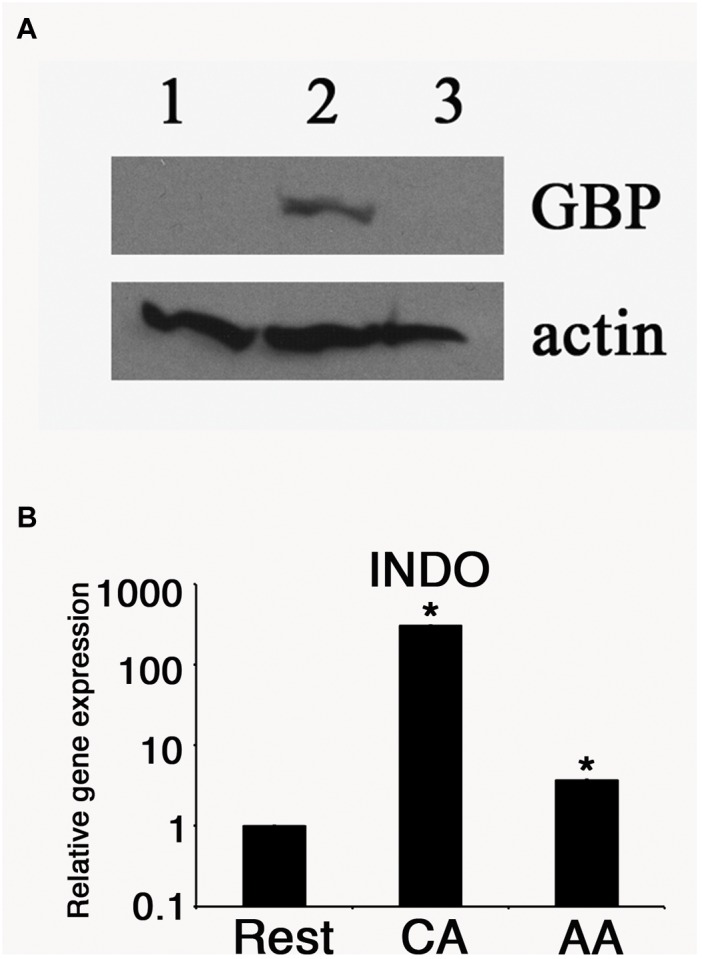
Antimicrobial effectors GBP and IDO are reduced in AA mϕ. **(A)** Immunoblots specific for human GBP-1 of resting mϕ (1), CA mϕ (2), or AA mϕ (3) are shown together with the protein levels of actin (actin). **(B)** Relative mRNA expression for IDO (INDO) of resting mϕ (Rest), CA mϕ (CA), or AA mϕ (AA). Real time PCR data were statistically evaluated with group-wise comparison and Relative Expression Software Tool (Pfaffl et al., 2002). Statistically significant data are labeled with ^∗^*p* ≤ 0.001.

We then monitored the expression of host proteins that are known to influence chlamydial growth. We previously reported that human guanylate binding proteins (hGBP) potentiated the anti-chlamydial effects of IFN-γ. Therefore, we monitored the relative hGBP1 levels in resting, AA mϕ, and CA mϕ by Western blot. As shown in Figure 5, hGBP1 showed a strong band in the CA mϕ group, while it was undetectable in lysates from AA mϕ and resting mϕ.

The expression of another anti-chlamydial effector, IDO was monitored in the same cell types. Total RNA was harvested from each samples, and the levels of IDO mRNA were quantified by reverse transcriptase-quantitative polymerase chain reaction (RT-qPCR). The relative expression was 1 (±0.005) for resting macrophages, 328. 6 (±1.15) for CA mϕ, and 3.6 (±0.01) for AA mϕ (Figure 5). We observed a 91-fold less IDO expression in AA mϕ relative to CA mϕ. Resting mϕ also exhibited a comparably low level of IDO transcripts, with a 328-fold decrease relative to CA mϕ.

**FIGURE 6:**
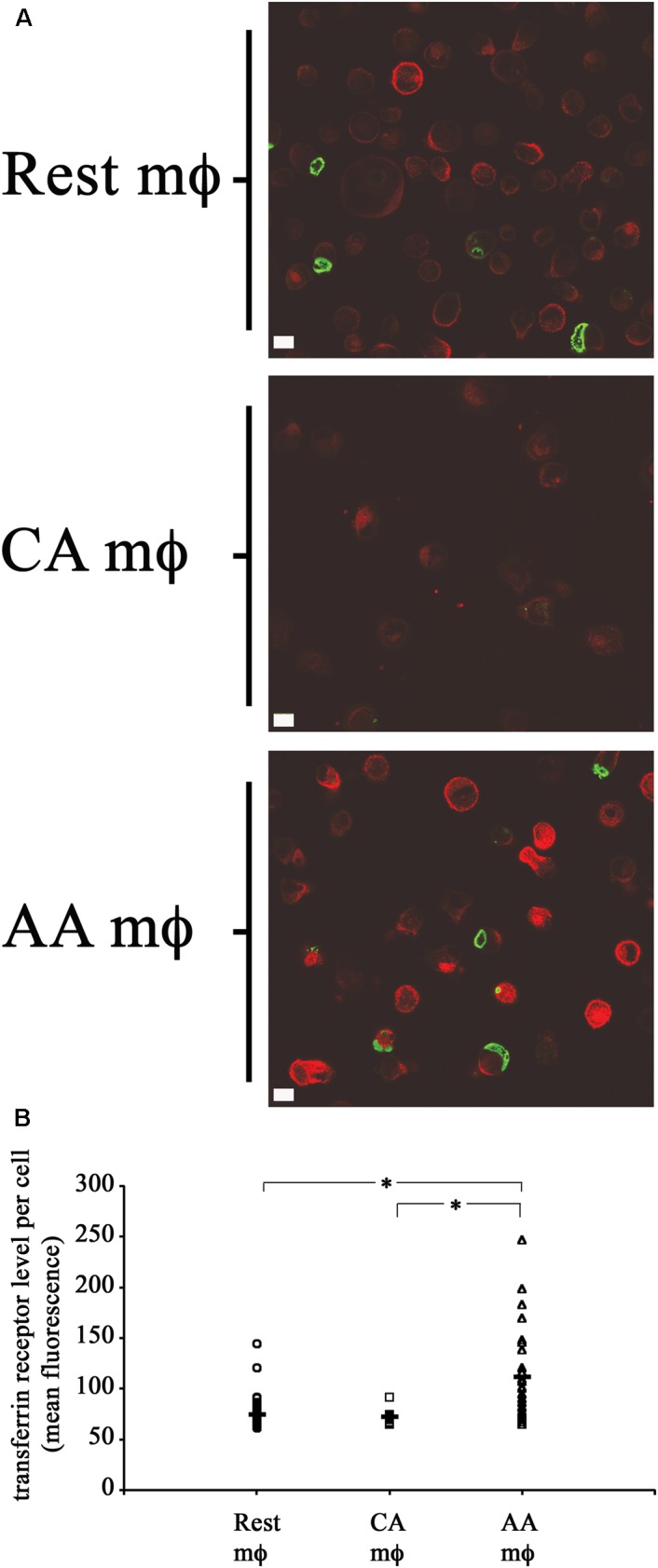
High transferrin receptor (TfR) level and growth of chlamydial inclusions in AA mϕ, but not CA cells. **(A)** Confocal micrographs of infected macrophages pulsed for 1 h with immunofluorescent transferrin (red). *Chlamydia trachomatis* was detected with indirect immunofluorescent antibodies (green). Detectable transferrin levels for AA mϕ > resting mϕ > CA mϕ. The white scale bar indicates 10 μm. **(B)** The Fiji release of ImageJ was used to analyze the levels of fluorescently labeled TfR of resting macrophages (Rest), classically activated (CA) and alternatively activated (AA) mϕ in micrographs obtained by confocal laser microscopy. The mean fluorescence of individual cells derived from the mean “gray” value of the red channel were computed in ImageJ. Scatter plots of individual resting macrophages (circular symbol, *n*: 58 cells), CA macrophages (square symbol, *n*: 13 cells), and AA macrophages (triangular symbol, *n*: 28 cells), together with the mean value for each group (horizontal bar) are depicted. The mean value for Rest mϕ was 74.51843, for CA mϕ was 72.11838, and for AA mϕ was 111.5917. One-way ANOVA analysis determined a statistically significant difference between the three groups with a *p*-value of 0.00000004429. A Tukey Honest Significant Difference *post hoc* test identified that the differences of TfR levels between AA mϕ and Rest mϕ are statistically significant, as well as between AA mϕ and CA mϕ with *p*-values below 0.0001 (symbolized as asterisk ^∗^). The TfR levels between CA mϕ and Rest mϕ was not found different in a statistically significant way.

The levels of transferrin receptor (TfR) in resting, AA mϕ, and CA mϕ were monitored. Ouellette and Carabeo demonstrated that chlamydial growth and replication depended on a functional slow recycling pathway of TfR (Ouellette and Carabeo, 2010), which might be involved in transporting iron to the chlamydial inclusion. The different macrophage types were pulsed for 1 h with transferrin that was conjugated to a fluorochrome. Unbound labeled transferrin was rinsed away, and samples and the levels of transferrin were visualized by confocal microscopy. As shown in Figure 6, TfR expression (red) was qualitatively higher in resting and AA mϕ samples relative to CA mϕ. Furthermore, the higher levels of TfR expression in these cell types correlated with the presence of larger inclusions of *C. trachomatis* (green).

Taken together, these results suggest that resting and AA mϕ are hospitable hosts to *C. trachomatis*, which may relate to their attenuated expression of known anti-chlamydial factors, e.g., hGBP1 and IDO, and the sustained high-level expression of TfR. Furthermore, *C. trachomatis* infection did not affect the expression of hallmark cell surface markers in either activated populations. However, infection appeared to influence the cell surface expression of all markers investigated in the resting mϕ population, which may be indicative of its uncommitted, pliable lineage.

### Differential Regulation of IDO Production in mϕ in Response to Viability and Type of Bacteria

**FIGURE 7:**
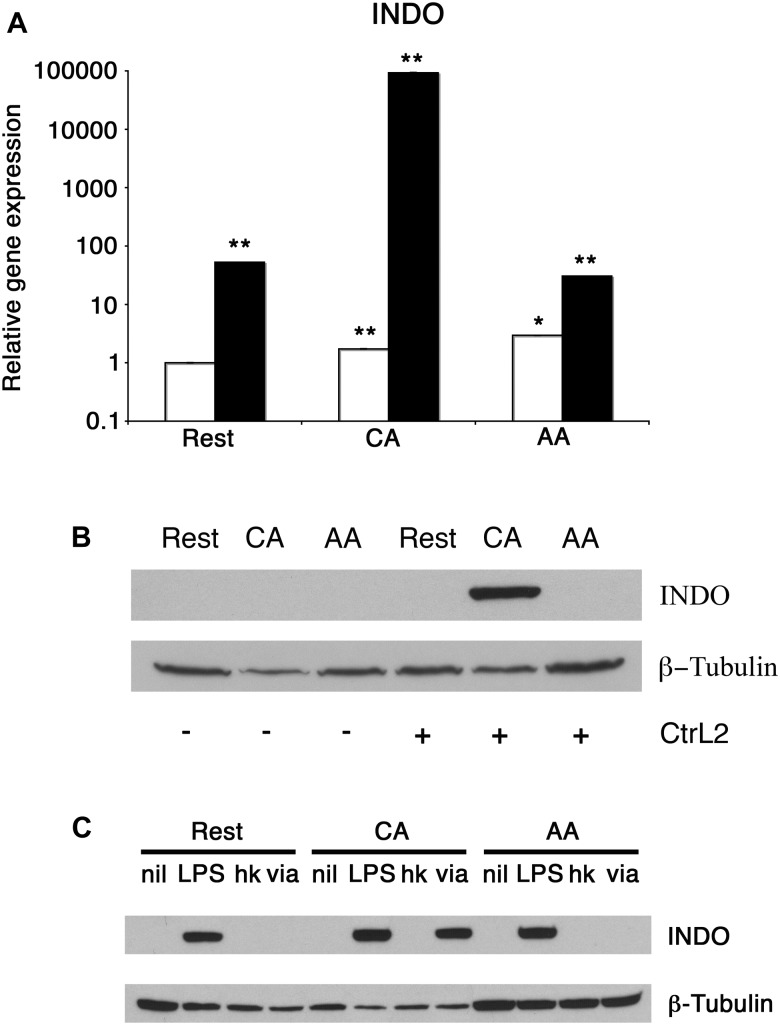
Differential induction of IDO protein in mϕ in response to infection and treatment. **(A)** Real-time PCR monitored mRNA expression of uninfected (empty bars) or infected (solid bars) mϕ types one day post-infection. ^∗∗^*p* ≤ 0.001; ^∗^*p* ≤ 0.035 for comparison to untreated resting mϕ using group-wise comparison and Relative Expression Software Tool (Pfaffl et al., 2002). **(B)** Western blots of resting macrophages (Rest), AA mϕ (AA), and CA mϕ without (-) exposure to or infected with (+) *Chlamydia trachomatis* L2 (CtrL2). **(C)** Immunoblots of protein levels of IDO1 (INDO) of mϕ left untreated (nil) or stimulated with *Escherichia coli* LPS (LPS), inoculated with heat-killed (hk) or viable (via) *Chlamydia trachomatis*. ß-Tubulin served as loading control.

We next determined the influence of *C. trachomatis* infection on anti-chlamydial effector molecules such as IDO. RT-PCR for IDO showed an increase of transcript for infected macrophages. The relative IDO expression for uninfected resting macrophages increased 53-fold with infection (Figure 7A). The relative IDO expression of uninfected CA mϕ increased 54682-fold upon infection (1.7 vs. 92960). The relative IDO expression for uninfected AA mϕ increased 10-fold compared to infected cells (2.9 vs. 30.6). To confirm this up-regulation of transcript translates into protein levels, Western blots of IDO were performed. Immunoblots did show detectable IDO only in infected CA mϕ, but not in resting or AA mϕ (Figure 7B). The question then arose whether heat-stable components of *C. trachomatis* or viable *C. trachomatis* are sufficient to induce IDO protein in macrophages and how it compares to treatment with lipopolysaccharide (LPS) from *Escherichia coli* (Figure 7C). Interestingly, only viable but not heat-killed *C. trachomatis* was able to induce IDO. Furthermore, *E. coli* LPS – unlike *C. trachomatis* – led to detectable IDO in not only CA, but also resting and AA mϕ (Figure 7C).

### *Chlamydia trachomatis* Infection of AA mϕ Impacts Its Production of IL-10

That *C. trachomatis* survived and replicated in AA mϕ raised the possibility of pathogen modulation of AA mϕ function. In this regard, we considered that infection would lead to potential changes in cytokine production. In the following experiments, we monitored two major cytokines with opposing effects – IL-10 and IL-12p70, which are anti- and pro-inflammatory cytokines, respectively.

**FIGURE 8:**
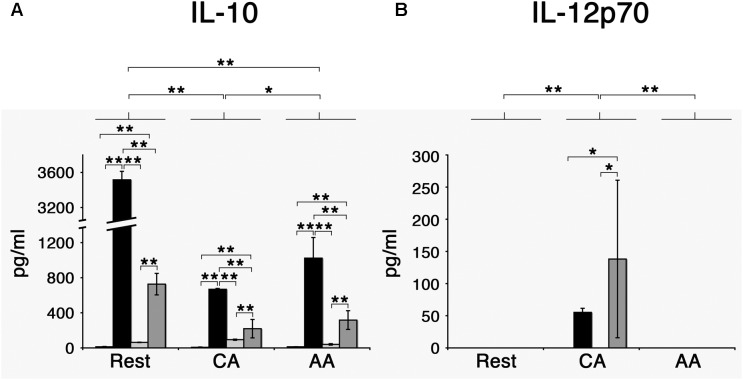
*Chlamydia trachomatis* induces IL-10 production in AA mϕ, and IL-12p70 in CA mϕ. **(A)** Mϕ were left untreated (first bar) or stimulated with *Escherichia coli* LPS (second bar), inoculated with heat-killed (third bar) or viable *Chlamydia trachomatis* (fourth bar) for one day. IL-10 concentrations of these cell culture supernatants were measured. Means of triplicates with respective standard deviations are depicted. After two-way ANOVA analysis, *p*-values of *post hoc* Tukey HSD are depicted. *p*-values ≤ 0.001 are symbolized by ^∗∗^. *p*-values ≤ 0.05 are symbolized by ^∗^. Results representative of one out of four independent experiments are shown. **(B)** Results for IL-12p70. *p*-values ≤ 0.01 are symbolized by ^∗∗^, *p*-values ≤ 0.5 are symbolized by ^∗^. Results representative of one out of four independent experiments are shown.

When stimulated with *E. coli* LPS, AA mϕ responded by producing high amounts of anti-inflammatory IL-10 in contrast to CA mϕ (mean value of triplicates of 1024 pg/ml with standard deviation of 235 pg/ml vs. mean value of 669 pg/ml with standard deviation of 9 pg/ml) (Figure 8).

Two-way analysis of variance (ANOVA) analysis for the cytokine production of IL-10 determined statistically significant differences for the type of treatment (mock, LPS, heat-killed Chlamydia, and viable Chlamydia) with a *p*-value of <0.001, for the type of macrophages (resting, CA, and AA macrophages) with a *p*-value of <0.001, and the interaction of treatment and type of macrophages with a *p*-value of <0.001. *Post hoc* analysis using TukeyHSD determined that the type of macrophages was a statistically significant factor in the IL-10 response. The differences between resting mϕ and AA mϕ were significant (*p*-value <0.001), the differences between resting mϕ and CA mϕ were significant (*p*-value < 0.001), and the differences between CA mϕ and AA mϕ were significant (*p*-value of 0.034). *Post hoc* analysis using TukeyHSD determined that the type of treatment was a statistically significant factor in the IL-10 response for most conditions. The differences between treatment with LPS and treatment with heat-killed *C. trachomatis* were significant (*p*-value < 0.001), the differences between treatment with viable *C. trachomatis* and treatment with heat-killed *C. trachomatis* were significant (*p*-value < 0.001), the differences between mock treatment and treatment with LPS were significant (*p*-value < 0.001), the differences between treatment with viable *C. trachomatis* and treatment with LPS were significant (*p*-value < 0.001), and the differences between treatment with viable *C. trachomatis* and mock treatment were significant (*p*-value < 0.001). Only the differences between treatment with heat-killed *C. trachomatis* and mock treatment were not significant (*p*-value of 0.6).

However, the same treatment elicited the opposite effect with regards to IL-12p70 production. LPS-stimulated CA mϕ produced many-fold higher level of the IL-12p70 compared to AA mϕ (mean value of 55 pg/ml with standard deviation of 6 pg/ml vs. undetectable levels). The effects of *C. trachomatis* infection on the profile of IL-10 and IL-12p70 production were investigated, with heat-killed EBs of *C. trachomatis* used as control. First, we observed that infection with viable *C. trachomatis* elicited stronger responses from AA mϕ and CA mϕ relative to exposure to heat-killed *C. trachomatis*. Infection of AA mϕ with viable bacteria led to a 7.7-fold higher level of IL-10 than inoculation with heat-killed bacteria (mean value of 317 pg/ml with standard deviation of 107 pg/ml vs. mean value of 41 pg/ml with standard deviation of 8 pg/ml). IL-12p70 production was many-fold higher in CA mϕ infected with viable *C. trachomatis* compared to inoculation with heat-killed EBs (mean value of 138 pg/ml with standard deviation of 122 pg/ml vs. undetectable levels). The respective responses of AA mϕ and CA mϕ to *C. trachomatis* infection were generally lower in magnitude relative to *E. coli* LPS treatment, the positive control. In this group, IL-10 production by AA mϕ (mean value of 1024 pg/ml with standard deviation of 235 pg/ml vs. mean value of 317 pg/ml with standard deviation of 107 pg/ml) and CA mϕ (mean value of 669 pg/ml with standard deviation of 9 pg/ml vs. mean value of 220 pg/ml with standard deviation of 105 pg/ml), respectively were 3.2- and 3.04-fold greater when compared to infection with viable *C. trachomatis*.

Two-way ANOVA analysis for the cytokine production of IL-12 determined statistically significant differences for the type of treatment (mock, LPS, heat-killed Chlamydia, and viable Chlamydia) with a *p*-value of 0.039, for the type of macrophages (resting, CA, and AA macrophages) with a *p*-value of 0.0030, and the interaction of treatment and type of macrophages with a *p*-value of 0.0142. *Post hoc* analysis using TukeyHSD determined that CA mϕ were significantly different from resting mϕ (*p*-value of 0.0073), and that CA mϕ were significantly different from AA mϕ (*p*-value of 0.0073). AA mϕ were not significantly different from resting mϕ (*p*-value of 1). In regard to treatment, differences between treatment with viable *C. trachomatis* and heat-killed *C. trachomatis* was significantly different (*p*-value of 0.0498189). Also the differences between mock treatment and viable *C. trachomatis* was significantly different (*p*-value of 0.0498189).

Taken together, the data indicate that *C. trachomatis*, either viable or heat-killed, is less potent in inducing changes to IL-10 and IL-12p70 production by AA mϕ and CA mϕ, respectively, compared to *E. coli* LPS. This may reflect the differences in the structure of the Lipid A portion between the chlamydial and *E. coli* LPS. However, IL-10 production was greater in infected AA mϕ, which raises the possibility that *C. trachomatis* may be actively promoting the high expression of this anti-inflammatory cytokine.

### Effects of IL-10 on Inclusion Development of *Chlamydia trachomatis*

**FIGURE 9:**
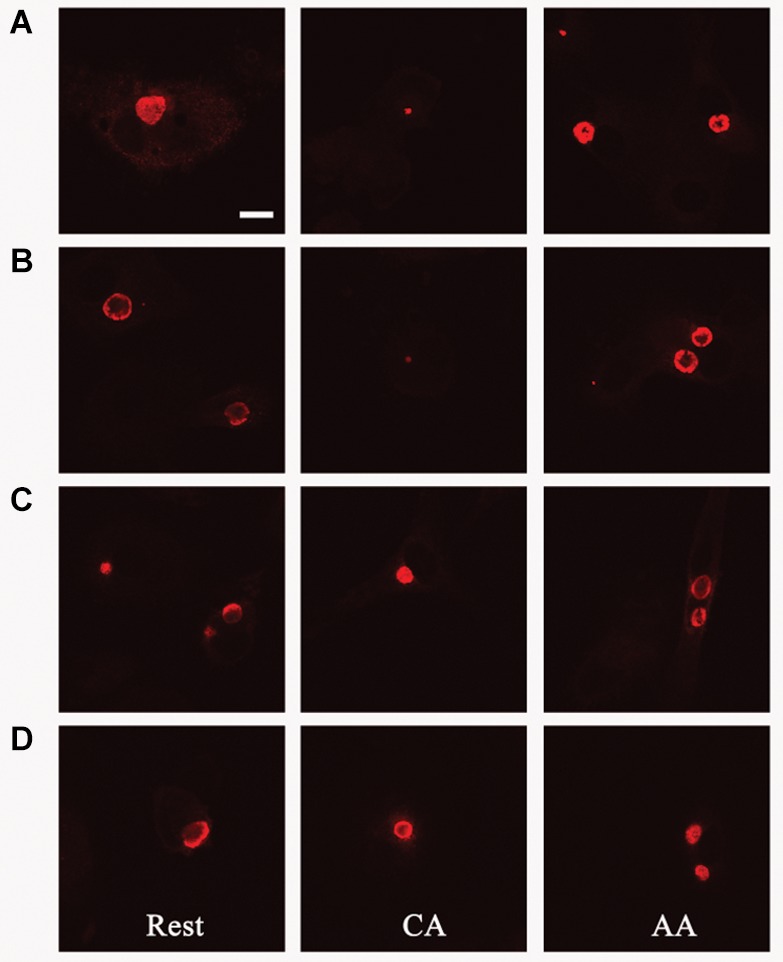
Pre-treatment with IL-10 overcomes growth restriction of chlamydia in CA mϕ. Fluorescently labeled chlamydial inclusions of human macrophages 18 to 24 h post-infection are shown **(A–D)**. Macrophages were treated with human interleukin-10 at 48 h **(D)**, at 24 h **(C)** before infection, simultaneously with infection **(B)**, or not treated at all with IL-10 **(A)**.

**FIGURE 10:**
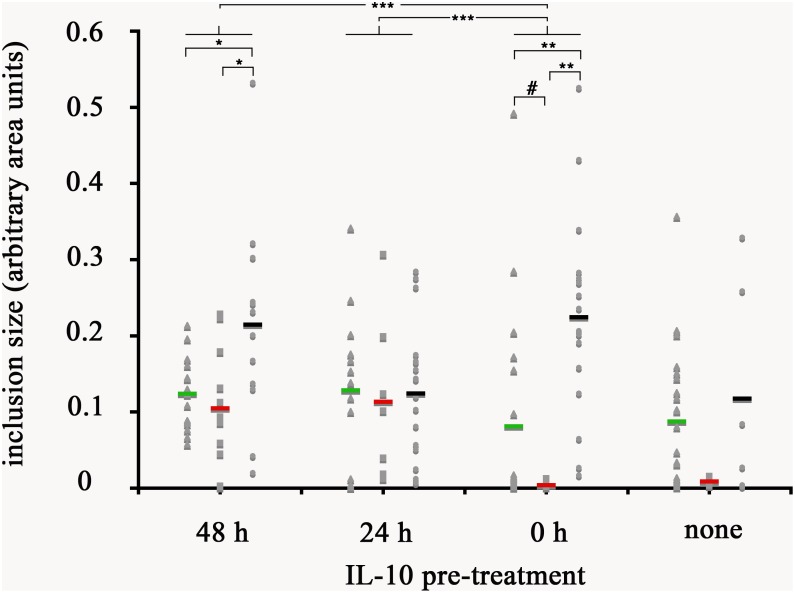
Quantification of inclusion size of mϕ without and with reversal of growth restriction following IL-10 pre-treatment. The photomicrographs were analyzed with ImageJ for size of individual inclusions, graphed in Microsoft Excel, followed by statistical analysis. Macrophages were treated with human interleukin-10 at 48 h (48 h), at 24 h (24 h) before infection, simultaneously with infection (0 h), or not treated at all with IL-10 (none). Resting macrophages are symbolized by circles, CA mϕ by squares, and AA mϕ by triangles. Means are indicated by horizontal bars. Inclusion size for CA mϕ for 0 h are different than 48 h with statistical significance. *p*-values equal or less than the significance of 0.1 are symbolized by (#), *p*-values ≤ 0.05 by one asterisk (^∗^), *p*-values ≤ 0.01 are labeled (^∗∗^), and *p*-values ≤ 0.002 are referenced as (^∗∗∗^).

Given that IL-10 production by AA mϕ could be modulated by *C. trachomatis*, we first investigated the effects of this cytokine on the ability of resting mϕ, AA mϕ, and CA mϕ to support chlamydial inclusion development. Resting mϕ, CA mϕ, or AA mϕ were pretreated with IL-10 for 48 h, 24 h before infection with *C. trachomatis* or simultaneously at time of infection. Mock-treated controls were included. Inclusion development was monitored by confocal microscopy, and inclusion diameter measured using NIH ImageJ (Figure 9, 10). We observed that the ability of resting mϕ and AA mϕ to support chlamydial growth was not significantly affected by either pre-treatment or simultaneous treatment with IL-10 relative to the timing of infection. Remarkably, CA mϕ exposed to IL-10 via either treatment protocol exhibited larger inclusions when compared to the mock-treated controls, indicating that the anti-chlamydial function was dampened by IL-10. Confocal microscopy showed strong reversal of growth restriction of *C. trachomatis* in CA mϕ pretreated with IL-10 for 24 or 48 h. The mean inclusion size for CA mϕ without IL-10 was 0.0088 arbitrary units with a standard deviation of 0.0058 and 0.0037 arbitrary units with a standard deviation of 0.0034 for treatment at time of infection. The mean inclusion size with 24 or 48 h of IL-10 pre-treatment was 0.1132 arbitrary units with a standard deviation of 0.1039 and 0.1044 arbitrary units with a standard deviation of 0.0758, respectively. Inclusions were generally 12-fold larger in the IL-10-exposed CA mϕ. Increased duration of pre- or co-treatment with IL-10 also correlated with increasing inclusion size, suggesting that IL-10 continued to dampen growth restriction of CA mϕ on *C. trachomatis*. One way ANOVA analysis based on *F* test statistics concluded that the resting, AA and CA mϕ groups were not all the same at 0 h treatment. The *post hoc* Tests, the multiple comparisons using LSD below showed that the differences for resting vs. CA and resting vs. AA mϕ are significant at the level 0.01 (^∗∗^) and CA vs. AA mϕ are significant at the level 0.1 (#). The pairwise comparisons with Bonferroni tests on treatments determined that 0 h vs. 24 h (significance of 0.002; (^∗∗∗^)) groups and 0 h vs. 48 h groups (significance of 0.002; (^∗∗∗^)) are significantly different.

### AA mϕ Revert Growth Restriction of *Chlamydia trachomatis* in Bystander CA mϕ

**FIGURE 11:**
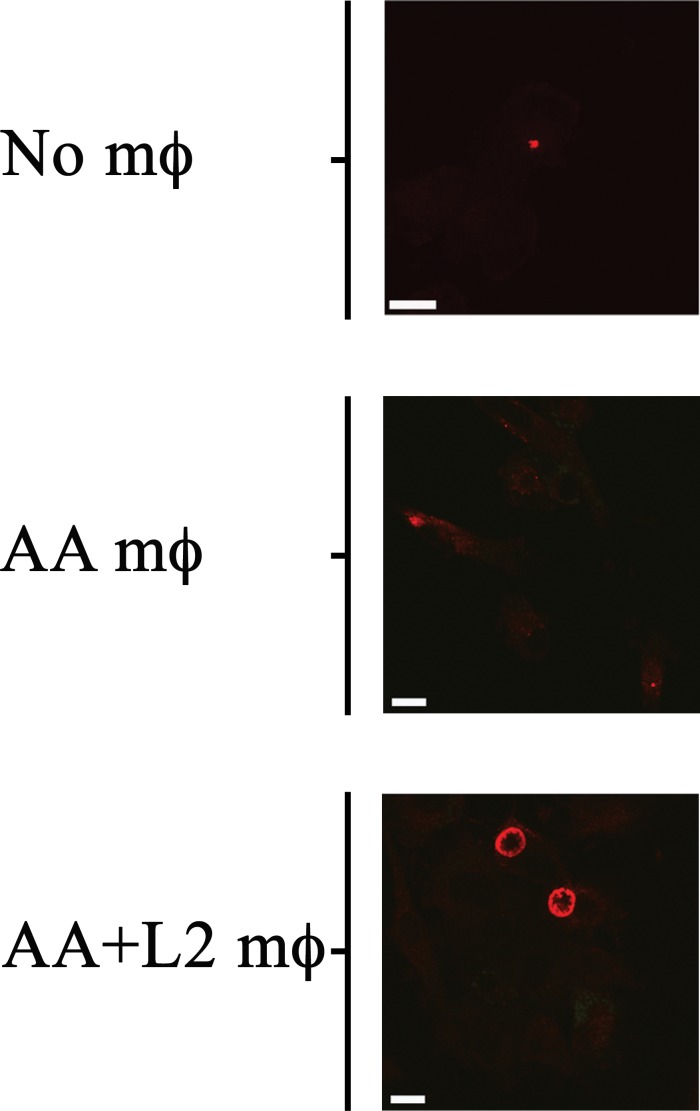
Bystander AA mϕ revert growth inhibition of *Chlamydia trachomatis* in CA mϕ. Fluorescently labeled chlamydial inclusion(s) inside CA mϕ of bottom wells co-cultured with no AA mϕ in top inserts (No mϕ), with uninfected AA mϕ in top inserts (AA mϕ), or with infected AA mϕ in top inserts (AA+ L2 mϕ). The scale bar indicates 10 μm.

**FIGURE 12:**
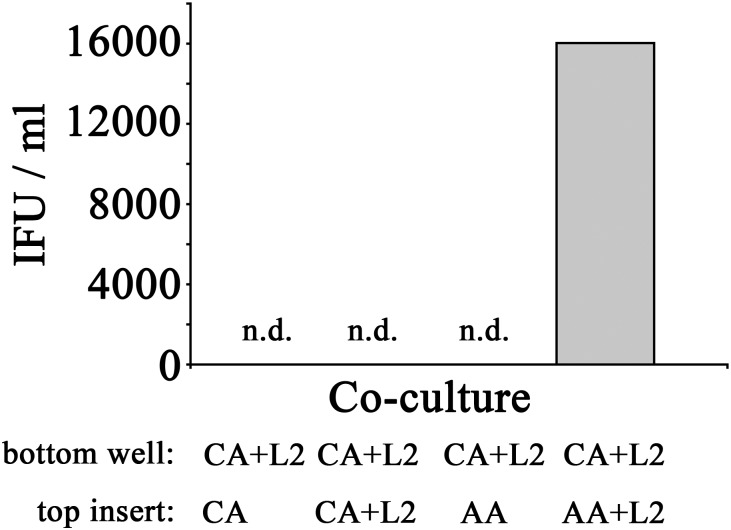
Recovery of inclusion forming units of CA mϕ reverted with bystander AA mϕ. Infected CA mϕ of bottom wells (CA+L2) were co-cultured with top inserts containing uninfected CA mϕ (CA), infected CA mϕ (CA+L2), uninfected AA mϕ (AA), or infected AA mϕ (AA+L2). After 24 h of co-culture, IFUs were recovered and used to infect HeLa cells. Undetectable levels of recovered IFU are indicated as n.d.

Since AA mϕ were shown to secrete paracrine IL-10 after *C. trachomatis* infection, and IL-10 could reverse the growth restriction of *C. trachomatis* in CA mϕ, we next investigated if AA mϕ could reverse growth restriction of *C. trachomatis* in CA mϕ in co-culture experiments. CA mϕ were infected in transwell experiments and cultured by itself, or in combination with uninfected AA mϕ, or in combination with infected AA mϕ (Figure 11, 12). Confocal microscopy showed growth of *C. trachomatis* inclusions in CA mϕ when infected bystander AA mϕ were present.

In order to assess if the reversal of growth inhibition in CA mϕ by AA mϕ led to a completed development cycle of *C. trachomatis* with newly made infectious EBs, IFUs from CA mϕ were recovered. Uninfected CA mϕ, infected CA mϕ, or CA mϕ with uninfected AA mϕ showed no detectable levels of IFU for extract from CA mϕ. In contrast, CA mϕ that showed reversal of growth inhibition when co-cultured with infected AA mϕ showed substantial amounts of recovered IFUs (Figure 12).

## Discussion

We hypothesized that alternatively activated macrophages would allow intracellular growth of *C. trachomatis*. To our knowledge this type of macrophage has not been studied as a potential host cell for *C. trachomatis*. Since alternatively activated macrophages are found during tissue repair, and infections with *C. trachomatis* induces both tissue damage and ensuing tissue repair, we determined it an important population to study.

The primary human macrophages of the resting, classically or alternatively activated phenotype we generated *in vitro* displayed features consistent with established characterizations (Gordon, 2003; Buchacher et al., 2015). Our AA mϕ (Figure 1, 6) expressed high levels of CD206, low levels of CD64 and high levels of TfR in comparison to classically activated mϕ (Kahnert et al., 2006). Furthermore, AA mϕ produced more anti-inflammatory IL-10 in response to stimulation with *E. coli* LPS than CA mϕ. Conversely, CA mϕ produced more inflammatory IL-12p70 (Figure 1). Consistent with the reduced inflammatory responses of AA mϕ, the *in vitro* generated untreated AA mϕ showed higher mRNA levels of anti-inflammatory IL-1 receptor antagonist and TGF-beta than CA mϕ (Rappolee et al., 1988; Goerdt et al., 1999). It was reported, that a combination of IL-4, IL-10, and TGF- β induce the most stable immunosuppressive phenotype of alternatively activated macrophage (Mia et al., 2014). We also observed characteristics in alternatively activated macrophages that suggested suitability as hosts to intracellular pathogens, such as *C. trachomatis*. First was the increased production of IL-10 in response to LPS (Figure 1). Second was the increased expression of TfR (Figure 6), which Ouellette and Carabeo (2010) have proposed to be involved in vesicle-mediated delivery of iron to the inclusion. Further, alternative activation did not induce the protein expression of IDO (Figure 7) or hGBP-1 (Figure 5), which has been shown to have potent anti-chlamydial activity. Indeed, the apparent attenuation of the anti-microbial arsenal of macrophages when alternatively activated reflected the observed inclusion development, chlamydial replication, and the completion of the biphasic developmental cycle. Therefore, we conclude that alternatively activated macrophages are suitable hosts for *C. trachomatis*.

Interestingly, our observations suggested that *C. trachomatis* initiates and possibly maintains this attenuated microbicidal character of alternatively activated macrophages. Infection of both resting and IL-4 activated macrophages induced the production of IL-10, thus raising the possibility that this cytokine acts in an autocrine manner to maintain the alternative activation phenotype, or induces it in the case of resting macrophages. IL-10 transcriptional regulation can involve several transcription factors such as STAT3 (Benkhart et al., 2000), NF-kappaB (Cao et al., 2006), CREB (Alvarez et al., 2009), Sp1 (Ma et al., 2001), c-Maf (Cao et al., 2005) and promoter remodeling (Zhang et al., 2006). The mechanism of activation associated with *C. trachomatis* infection is not known, but there is precedent for *C. trachomatis* modulating the activity of specific transcription factors. A recent example demonstrated the modulation of STAT1 phosphoactivated in response to IFN-γ. Ibana et al. (2018) reported that in infected endocervical epithelial cells, phosphorylated STAT1 (pSTAT1) did not translocate to the nucleus, in contrast to observations in neighboring uninfected cells. Transcription mediated by the AP-1- transcription factor was also enhanced in *C. trachomatis* -infected HeLa cells through the indirect action of *C. trachomatis* via the host proteins Pin1 and Men1, which are known AP-1 regulators (Olive et al., 2014). We propose that the enhanced IL-10 production is actively driven by *C. trachomatis* based on our observation that alternatively activated macrophages produced more IL-10 when infected with viable *C. trachomatis* compared to macrophages inoculated with heat-killed bacteria.

Another intriguing observation we made was the effect of IL-10 produced by infected alternatively activated macrophages on the ability of nearby classically activated macrophages to suppress chlamydial development. Using a co-culture model, we found that infection in classically activated macrophages progressed to generate infectious particles in the presence of IL-10-producing *C. trachomatis*-infected alternatively activated macrophages. While we cannot conclusively attribute this function to IL-10, attenuated anti-microbial functions in classically activated macrophages are consistent with such effects of IL-10 on this macrophage subtype (Gordon, 2003). IL-10 was shown to deactivate macrophages which were treated with IFN-γ and LPS (Bogdan et al., 1991). Anti-microbial hydrogen peroxide and inflammatory cytokines such as TNF-α were reduced. Macrophages constitutively overexpressing IL-10 caused higher bacterial loads and reduced amounts of IL-12 and TNF-α (Lang et al., 2002). And mechanisms were described whereby microbes induce pro-inflammatory M1 macrophages to switch to an alternatively activated M2 phenotype that involved IL-10 (Plüddemann et al., 2011). Attenuation of bactericidal functions of neighboring classically activated macrophages could promote a more hospitable environment for *C. trachomatis*. Not only would it inhibit the propagation of a pro-inflammatory, but suppression of classically activated macrophage function would also increase the number of cells that can support *C. trachomatis* growth and development.

Interleukin-10 produced by infected alternatively activated macrophages might also affect resident dendritic cells. Exposure to IL-10 impairs the ability of dendritic cells to induce a Th1 response *in vivo* (Igietseme et al., 2000; Marks et al., 2010). These dendritic cells promoted the development of a Th2-like response, which is less efficient in resolving *C. trachomatis* infection (Su et al., 1998; Moniz et al., 2009; Ryans et al., 2017).

Taken together, the ability of *C. trachomatis* to infect, survive, and replicate in alternatively activated macrophages includes induction of IL-10 production. This could be essential in attenuating the anti-microbial functions of nearby classically activated macrophages, thus effectively increasing the number of cells in the vicinity to serve as potential hosts for subsequent rounds of infection. In addition, IL-10 production may also affect dendritic cells by suppressing their capacity to induce a Th1 pro-inflammatory response (Igietseme et al., 2000; Marks et al., 2010).

Infection of resting, CA and AA mϕ showed, that *C. trachomatis* can grow in the AA mϕ type when compared to CA mϕ and indicated by the growth of *C. trachomatis* inclusions (Figure 2, 3). Interestingly, AA mϕ showed high expression of TfR (Figure 6). The high expression of TfR could conceivably increase the intracellular level of iron via import of transferrin-iron complexes. Limitation of iron availability is a host mechanism to control growth of intracellular pathogens (Forbes and Gros, 2001; Nairz et al., 2018). Increased TfR expression and iron import was implicated as beneficial for growth of *Mycobacterium tuberculosis* in alternatively activated macrophages (Kahnert et al., 2006). It is intriguing to consider the increased TfR expression contributing to survival of *C. trachomatis* by providing sufficient amounts of essential iron. The importance of iron for *C. trachomatis* is of renewed research interest (Pokorzynski et al., 2017; Brinkworth et al., 2018).

The recovery of inclusion forming units (IFUs) from AA, CA and resting macrophages showed significantly higher titers of IFUs for AA than for CA mϕ (Figure 3). These data are consistent with the confocal micrographs that showed robust chlamydial growth detected as inclusion size of infected macrophages. Taken together, the data suggest that *C. trachomatis* can infect, grow and complete its developmental cycle in alternatively activated (AA) mϕ in contrast to CA mϕ.

Future research could elucidate the complex interactions of different macrophage phenotypes during infection with *C. trachomatis*. The interactions of AA mϕ on CA mϕ were reported, but interactions of bystander mϕ on resting mϕ or other phenotypes remains to be elucidated. Bystander macrophages of the resting, AA, and CA phenotype had a different and more complex impact on resting macrophages (Supplementary Figure S1).

## Materials and Methods

### Isolation of Monocytes From Peripheral Blood

Peripheral blood from healthy donors was collected in 10 ml Vacutainer test tubes from (Beckton Dickinson) containing sodium heparin. Blood was diluted with equal volume of cell culture medium RPMI 1640 supplemented with 10% FCS, 2 mM L-glutamine and 10 μg/ml gentamycin. PBMC were separated from erythrocytes, polymorphonuclear cells (PMN) by centrifugation with Ficoll-Paque layer. For that purpose, 25 ml diluted blood was overlayed in 50 ml tubes onto an equal volume of endotoxin-tested sterile Ficoll-Paque Premium (GE-Health care) and centrifuged for 20 min at 450 *g* without forced deceleration (brakes). About 10 ml autologous human serum was retrieved from each 50 ml tube for later cell culture supplementation. The interphase with PBMCs was pooled and washed by adding phosphate-buffered saline (PBS) supplemented 0.5% FCS to a total volume of 50 ml. After centrifugation at 450 *g* for 10 min, the cell pellet was resuspended in 2–3 ml RBC lysis buffer (eBioscience). Incubation on ice for 10 min was sufficient to lyse residual erythrocytes. The remaining mononuclear cells were washed with about 30 ml 0.5% FCS in PBS. The cell pellet was resuspended in ample amounts (40 ml) of 0.5%FCS/PBS for a second washing step and cells were counted for the ensuing magnetically activated cell sorting (MACS). CD14-positive monocytes were positively selected via MACS from mononuclear cells according to the manufacturer’s instruction and as described by others (Kzhyshkowska et al., 2006). Fc-gamma receptors were blocked by adding Fc-blocking reagent (Miltenyi) to PBMCs. In brief, after an incubation time of 20 min on ice, CD14 MicroBeads (Miltenyi) were added. After 30 min on ice, cells were washed with 0.5%FCS/PBS and centrifuged at 450 *g* for 10 min at 4 °C. Using LS columns with the MidiMACS separator unit and the MACSMultiStand (Miltenyi) CD14-positive monocytes were separated from other PBMCs. Approximately 1 × 10^6^ monocytes were plated in 2 ml RPMI 1640 supplemented with 10% FCS, 10% human serum, 2 mM L-glutamine, 10 μg/ml gentamycin and 10 ng/ml M-CSF (referred to as M10 medium) into each well of a six-well tissue culture plate (Corning Costar). Typically, 10–20% of the PBMCs were monocytes.

### Ethics Statement

Peripheral blood from healthy donors was collected in 10 ml Vacutainer test tubes from (Beckton Dickinson) containing sodium heparin. The protocol was approved by the Institutional Review Board of Louisiana State University Health Sciences Center, New Orleans. All subjects gave written informed consent in accordance with the Declaration of Helsinki.

### Differentiation of Monocytes to Macrophages

Monocytes were differentiated into macrophages using M-CSF. In brief, 1 × 10^6^ monocytes were plated per well of a 6-well plate with 2 ml RPMI 1640 supplemented with 10% FCS, 10% human serum, 2 mM L-glutamine, 10 μg/ml gentamycin and 10 ng/ml M-CSF. Three days after plating, 2 ml of the same cell culture medium was added. Five days after cultivation, supernatants and non-adherent cells were removed. The adherent macrophages were used after an additional period of 2 days for experiments detailed below.

### Activation of Macrophages

Alternatively or classically activated macrophages were generated by using human interleukin (IL)-4 or gamma-interferon (IFN-γ), respectively, similar to published procedures (Bonecchi et al., 1998). In brief, macrophages were alternatively activated for 2 days *in vitro* using 100 ng/ml human recombinant interleukin (IL)-4 (R&D systems cat #204-IL-050/CF; endotoxin < 1.0 EU per 1 μg of the cytokine as determined by the LAL method). In parallel, classically activated macrophages were generated by using 10 ng/ml human recombinant gamma-interferon (IFN-γ) (R&D systems). Untreated macrophages served as a control for resting macrophages. The different types of macrophages were characterized by their expression of surface markers and the cytokine response to LPS of Gram-negative bacteria. In our hands, the macrophages were positive for the macrophage marker CD14 and negative for the marker of dendritic cells, CD1/CD83. The expression levels for classically activated (CA) macrophages were CD64high, CD206 low, CD86low and TfR low. Alternatively activated (AA) macrophages (mϕ) were CD64 low, CD206 high, CD86 intermediate, TfR (high). Expression levels for resting mϕ were CD64 intermediate, CD206 intermediate, CD86 low or negative, TfR intermediate. In regard to the cytokine profile, AA mϕ IL-10 high and IL-12p70 low responders compared to classically activated macrophages that produced more IL-12p70 and less IL-10 to stimulation with *E. coli* LPS. The phenotype of CA and AA mϕ was consistent with published characterizations for human and murine cells (Gordon, 2003; Kahnert et al., 2006). Macrophages tested negative for CD1a, a marker of dendritic cells and positive for CD14.

### Indirect Immunofluorescence Assays (IFA)

Established protocols for intracellular detection of *C. trachomatis* after infection of HeLa cells were used with slight modifications (Carabeo et al., 2002, 2003).

In brief, 1 or 2 × 10^5^ macrophages were seeded onto sterilized and roughened 12 mm glass coverslips placed in 24-well plates. At the endpoint of the experiment, cells were fixed for 1 h at ambient temperatures with 1 ml 4% paraformaldehyde in PBS, followed by several washing steps with PBS. Cells were permeabilized for 2 to 4 min at ambient temperatures with 0.5 ml 0.1% Triton X-100 in PBS. After washing, cells were incubated with a primary rabbit polyclonal antibody recognizing EBs of *C. trachomatis L2*. The antibody was used at a 1:1000 dilution in 0.25 ml PBS per coverslip of 24-well plate and incubated for at least 1 h at ambient temperatures. After several washing steps with PBS, a secondary anti-rabbit monoclonal antibody conjugated to Alexa 594 was used at 1:1000 dilution in 0.25 ml PBS and added to the coverslip for 1 h. After several washing steps coverslips were mounted with 10 μl MOWIOL with the anti-fading reagent 5,5’-Dithiobis (2-nitro-benzoic acid) onto microscopic slides. Macrophages and *C. trachomatis* were visualized under 40 or 60-fold magnification with an Olympus Fluoview confocal microscope (Olympus, Pittsburgh, PA) equipped with an argon and HeNe-G laser (Daep et al., 2006). The shown images were normalized by using Adobe Photoshop and selecting input levels from 0 to 165.

### Infection of Macrophages With *Chlamydia trachomatis*

Macrophages were infected with purified EBs of *C. trachomatis L2* as described elsewhere (Carabeo et al., 2002, 2003). For that purpose, macrophages were inoculated with EBs at MOI of 1 in 0.25 ml HBSS or PBS for 30 min on ice unless noted otherwise. Alternatively, macrophages were inoculated with *C. trachomatis* using centrifugation for 10 min at 4 °C with 900 *g*. Afterward the inoculum was removed and replaced with fresh pre-warmed cell culture medium. The incubation was stopped about 20 h post-infection for IFA and cytokine assays as well as FACS analysis.

### Heat-Killed *Chlamydia trachomatis* and LPS Stimulation

Heat-killed *C. trachomatis* was prepared using a previously reported procedure (Kalayoglu and Byrne, 1998). *E. coli* LPS was used at a final concentration of 100 ng/ml.

### Recovery of Inclusion Forming Units (IFUs)

1 × 10^5^ macrophages per coverslip of a 24-well plate were infected with *C. trachomatis* (CtrL2) at a MOI of 1. After an infection period of 26 h cell culture supernatants were removed. The infected cells containing the EBs of *C. trachomatis* were lysed by incubation for 2 min in 1 ml of sterile water per well with repeated pipetting of the mixture. Thereafter, the lysates were transferred to sterile 1.5 ml tubes and sonicated in a water bath for additional 2 min at ambient temperatures. The liberated EBs were collected by a 15 min centrifugation with 20,000 *g* at 4 C. The supernatant was discarded and the EBs were resuspended in 0.25 ml PBS for each well of a 24-well plate. This stock was used to infect HeLa cells. In parallel, cells were inoculated with aliquots of dilution series, e.g., preferably 1:1, 1:10, 1:100 or 1:1, 1:5, and 1:25 series. To that effect 0.25 ml suspension of EBs in PBS were transferred to nearly confluent (>1 × 10^5^) HeLa cells on coverslips in 24-well plates. After 30 min on ice, the inoculum was removed and replaced with 1 ml cell culture medium per well. The incubation period was terminated 24 h post-infection and inclusions were assessed by using indirect immunofluorescence assays (IFA) with *C. trachomatis* -specific antibodies. IFU were counted manually with a fluorescence microscope. Alternatively, in most cases IFUs were documented using a confocal microscopy and photomicrographs were afterward counted manually using the cell counter plugin feature of the ImageJ software. The number of IFU was calculated with the following formula:

• IFU = [(number of inclusions)/(number of microscopic fields) × (CF) × (dilution factor)];
• whereby the conversion factor was defined as (CF) = [(total area of well)/(area of microscopic field of view)]

Unless noted otherwise, five optical fields for each treatment group and dilution was analyzed including the mean and standard deviation. A total of four similar experiments were performed. Four experiments used an MOI of one, one study used additionally a MOI of 5. The one-way of variance (ANOVA) in combination with a Dunnett multiple comparisons test was used for statistical analysis of IFU-data. The overall *p*-value and individual groups vs. the control group (i.e., resting macrophages) were calculated for each MOI separately. For that purpose, InStat software from GraphPad was applied.

### Co-culture Experiments

For co-culture experiments a transwell system with 6.5 mm top insert containing a permeable 0.4 μm polycarbonate membrane and appropriate 24-well tissue culture plate was used (Corning Costar). The permeable membrane prevented migration of macrophages but allowed soluble mediators like cytokines to pass through to the bottom well. 1 × 10^5^ mϕ were plated into the top insert and 2 × 10^5^ macrophages were seeded onto coverslips in the bottom wells in cell culture medium with M-CSF and 10% human serum. Unless otherwise noted, the activation of the different types of macrophages was done in separate bottom wells or inserts. After an activation of macrophages for 2 days (resting mϕ were left untreated, classically activated macrophages were activated with 10 ng/ml human IFN-γ, alternatively activated macrophages were cultivated with 100 ng/ml human IL-4), top inserts with cells were placed into the appropriate bottom wells containing cells leading to the different combinations of the needed macrophages types. The various activating media were removed beforehand and replaced with cell culture medium without IFN-γ or IL-4. All infections used an MOI of 1. Due to the fragile nature of the permeable membrane of the top insert, *C. trachomatis* was inoculated in 0.1 ml PBS on ice for 30 min for experiments requiring infection of macrophages of the top insert. For the bottom wells, 0.2 ml inoculum was used. Infection periods lasted about 20–24 h. Experiments with sequential primary infection of top inserts followed by secondary infection of bottom wells lasted about 20–24 h each.

### Detection of Transferrin Receptors

Viable cells were pulsed for 1 h with Alexa Fluor 594 conjugated to transferrin. In brief, macrophages were plated and grown on coverslips in 24-well plates at a density of 0.5 or 1.0 × 10^5^ cells/well. At the end of the cultivation period, cell culture supernatant was removed and replaced with 100 μl PBS with 1:100 dilutions of aqueous 5 mg/ml stock of human transferrin Alexa Fluor 594 (Molecular Probes). After 1 h of incubation at 37°C in 5% CO_2_ atmosphere, cells were repeatedly washed with PBS and fixed with 1 ml 4% paraformaldehyde in PBS as described in the IFA procedure. TfR was detected using confocal microscopy. Analysis of uninfected cells and macrophages infected with *C. trachomatis* showed that infection of macrophages did not interfere TfR detection. The depicted micrographs were normalized by using Adobe Photoshop and selecting input levels from 0 to 165.

### Flow Cytometry Detection of Macrophage Markers

Macrophages were characterized for surface expression of CD1a, CD64 (high affinity receptor for Fc-gamma), CD86, and CD206 (mannose receptor). Adherent macrophages were harvested by use of non-trypsinizing Cellstripper (Mediatech, Inc.) according to manufacturer’s instruction. Cells were washed and unspecific binding of fluorescent antibodies to Fc-gamma receptors was prevented by incubation with Fc-blocking reagent (Miltenyi) for 20 min on ice in staining buffer containing 2% FBS and 0.09% sodium-azide (BD Pharmingen). Isotype controls or specific antibodies were then added for 30 min on ice according to manufacturer’s recommendations. Isotypes controls were FITC-conjugated mouse IgG1 (eBioscience), PE-conjugated mouse IgG1 (eBioscience), PE-conjugated mouse IgG2a (eBioscience), APC-conjugated mouse IgG2a (eBioscience), and APC-conjugated mouse IgG1 (eBioscience). The specific antibodies used for FACS were PE-conjugated CD1a (BD Pharmingen), APC-conjugated CD1a (BD Pharmingen), PE-conjugated CD64 (BD Pharmingen), APC-conjugated CD86 (BD Pharmingen), and FITC-conjugated CD206 (BD Pharmingen). After the incubation step a 10-fold volume of staining buffer was added to wash cells. After centrifugation with 450 *g* at 4°C the macrophages were resuspended in 0.4 ml to 1 ml staining buffer and analyzed with a FACSCalibur in combination with CellQuest Pro software. Typically, 10,000 gated events were acquired, analyzed and visualized as histogram. The MFI and standard deviation were calculated with the histogram statistics feature of CellQuest Pro, version 5.

### Reverse Transcription and Quantitative Real-Time PCR

Expression of human interleukin (IL)-4, IL-13, IL-1 receptor antagonist (IL-1Ra), transforming growth factor beta 1 (TGF-beta1) was assessed by isolating total RNA from macrophages followed by reverse transcription (RT) and quantitative real-time polymerase chain reaction (PCR). In more detail, 1 or 2 × 10^6^ mϕ per well were plated onto 6-well plates in a cell culture volume of 2 ml. At the endpoint of the experiment supernatant was removed and was analyzed in some cases for secreted cytokines using a cytometric bead assay (CBA). Cells were gently washed with 1 ml ice-cold PBS or DBPS. Cells were then lysed with 1 ml TRIZOL reagent (Invitrogen) for 5 min with repeated pipetting. Total RNA was isolated according to the manufacturer’s instructions. For the RT, the Superscript III Reverse Transcriptase system (Invitrogen) together with nonamer Random Primer 9 (New England Biolabs) was used according to product recommendations together with equal amounts of total RNA for different treatment groups, e.g., 500 ng (but no less than 200 or more than 1000). The resulting cDNA was diluted 2-fold with nuclease-free water (Ambion) and stored at –20 °C. For quantitative real-time PCR 3 to 5 μl of cDNA template, 2 μl of 10-fold concentrated specific primers and 12.5 μl of two-fold concentrated Power SYBR Green PCR Master Mix (Applied Biosystems) for a 25 μl reaction volume were used. The primers were obtained from Qiagen unless otherwise noted. The primers were for the gene IL-1RN cat #QT00014238 with a predicted amplicon length of 127 bp, for the gene TGFB1 cat #QT00000728 with a predicted amplicon length of 108 bp, for the gene IL13 cat #QT00000511 with a predicted amplicon length of 185 bp, for the gene IL4 cat #QT00012565 with a predicted amplicon length of 89 bp, and for the beta-actin gene ACTB cat #QT00095431 with a predicted amplicon length of 146 bp. The expression of the house-keeping gene beta-actin was used to normalize between different treatment groups. Samples were run in optical 96-well reaction plates (Applied Biosystems) sealed with optical adhesive film (Applied Biosystems) on an Applied Biosystems 7300 Real Time PCR System with the following protocol unless otherwise notified:

Stage 1 with 50°C for 1 min followed by 95°C for 10 min.

Stage 2 with 94°C for 15 s followed by 55°C for 30 s and 72°C for 35 s with data collection for continuous fluorescence reading, repeated 40 times.

Stage 3 with dissociation steps for melting curve analysis of specific and unspecific PCR products.

The end products of a PCR were analyzed on a 2% ethidium bromide agarose gel to verify amplification of a specific product with the corresponding predicted amplicon size. The relative expression of specific products was calculated and statistically evaluated using relative expression software tool from Pfaffl et al. (2002). Data are normally presented as means of triplicates with standard deviations as error bars. Some experiments had only duplicates. Three or more independent experiments were performed with similar results.

### Immunoblots of GBP-1 and IDO1

Guanylate Binding Protein-1 (GBP-1) was detected by immunoblotting as previously described (Tietzel et al., 2009). Instead of HeLa cells human macrophages were analyzed. Primary antibody was a rat anti-human GBP-1 from Oncogene, catalog number im-1011. As secondary antibody, a HRP-goat anti-rat IgG (H+L) Conjugate from Zymed or Pierce was used. For normalization, primary anti-actin, or anti-β-tubulin from Sigma were combined with secondary HRP-conjugated goat anti-mouse or anti-rat antibodies from Zymed or Pierce. *Homo sapiens* indoleamine 2,3-dioxygenase (INDO or IDO1) was detected with mouse anti-IDO antibody (Upstate, Clone 10.1) and a suitable secondary anti-mouse antibody from Pierce. Typically, lysates of at least 1 × 10^5^ macrophages were used.

### IL-10 Treatment of Macrophages

1 × 10^5^ macrophages were transferred into individual wells of a 24-well plate and infected with *C. trachomatis* at a MOI of 1. Macrophages were treated with human interleukin-10 at a final concentration of 10 ng/ml at 48 h (48 h), at 24 h (24 h) before infection, simultaneously with infection (0 h), or not treated at all with IL-10 (none). 18 to 24 h post-infection confocal laser microscopy of fluorescently labeled chlamydial inclusions was undertaken. The photomicrographs were analyzed with ImageJ for size of individual inclusions, graphed in Microsoft Excel, followed by statistical analysis.

### Cytokine Detection by Cytometric Bead Array (CBA)

Macrophage-secreted cytokines were analyzed by using a multiplex Cytometric Bead Array according to manufacturer’s instructions (BD CBA human inflammation kit cat #551811). In brief, 50 μl cell culture supernatant was mixed and incubated with equal amounts of cytokine-specific capture beads for IL-8, IL-1beta, IL-6, IL-10, TNF-alpha, IL-12p70 and detection reagent for 3 h at ambient temperatures without exposure to light. After washing with 1 ml of the provided buffer and centrifugation, the beads were resuspended in 0.3 ml buffer, run on a FACSCalibur and analyzed with the provided software. In a typical experiment, cell culture supernatants were taken 18 to 24 h post-infection or 100 ng/ml of LPS stimulation of macrophages that were plated at a density of 1 × 10^5^ cells per well of a 24-well plate. In some early experiments 1 × 10^6^ cells were plated in aliquots into 6-well plates.

### Statistical Analysis

The Mstat program kindly provided by Dr. Norman Drinkwater (University of Wisconsin-Madison) was used for statistical analyses^1^. To analyze differences in inclusion sizes of *C. trachomatis* between different treatment groups, a Wilcoxon test was used. *p*-values of less than 0.05 were presumed to be statistically significant.

For the statistical analysis of relative gene expression, the Relative Expression Software Tool from Pfaffl et al. (2002) was used. The pairwise allocation function was employed with 2,000 or 10,000 iterations and *p*-values lower than 0.05 were found to be statistically significant.

Cytokine data were analyzed with the GraphPad InStat software, version 3.0b or R version 3.5.2 (R Core Team, 2018). If appropriate groups were assayed with a one-way ANOVA test assuming parametric Gaussian distribution, under the condition of a multiple comparison posttest. The Dunnett post-test compared all groups vs. the control group, i.e., untreated resting macrophages (Rest nil). A *p*-value of less than 0.05 was assumed to be statistically significant. Data are presented as means of triplicates with standard deviations as error bars. If appropriate data were analyzed with a two-way ANOVA, followed by *post hoc* tests such as the Tukey Honest Significant Difference (HSD). For analysis of data groups compiled from different independent experiments, an ANOVA calculation with a Tukey–Kramer multiple comparisons test was executed. Overall and individual *p*-values were analyzed and found statistically significant if less than *p* ≤ 0.05.

For flow cytometric detection of macrophage markers the data were imported into the InStat program version 3 of GraphPad for assessment of statistical significance. The ANOVA analysis with the Dunnett multiple comparisons test calculated an overall *p*-value and individual *p*-values compared to the control group. The data were deemed statistically significant if values were less than 0.05. Data are representative of three or more similar independent experiments.

### Morphometric Analysis

Morphometric analysis of chlamydial inclusions was undertaken using ImageJ software^2^. Inclusions were marked with the Region of Interest (ROI) Manager tool function and analyzed by surface area. A Wilcoxon test of the Mstat program determined if differences of inclusion sizes (for different treatment groups) were statistically significant. Additionally, the data for inclusion size were re-analyzed with a one-way ANOVA using R software and appropriate *post hoc* tests including Bonferroni tests. The Fiji release of ImageJ was used to analyze the levels of fluorescently labeled TfR of the different macrophage types.

## Ethics Statement

Peripheral blood from healthy donors was collected in 10 ml Vacutainer test tubes from (Beckton Dickinson) containing sodium heparin. The protocol was approved by the Institutional Review Board of Louisiana State University Health Sciences Center, New Orleans. All subjects gave written informed consent in accordance with the Declaration of Helsinki.

## Author Contributions

IT conducted all experimental work. All authors contributed to the writing and review of the manuscript.

## Conflict of Interest Statement

The authors declare that the research was conducted in the absence of any commercial or financial relationships that could be construed as a potential conflict of interest.

## Footnotes

1 https://mcardle.wisc.edu/mstat/

2 http://rsb.info.nih.gov/ij/
