# Supplementary material for: Alternatively Activated Macrophages Are Host Cells for Chlamydia trachomatis and Reverse Anti-chlamydial Classically Activated Macrophages
Source: Front Microbiol. 2019 May 7;10:919. doi: 10.3389/fmicb.2019.00919 (PMC6524708; doi:10.3389/fmicb.2019.00919)
Supplement: Supplementary file 1 [file Image_1.pdf]

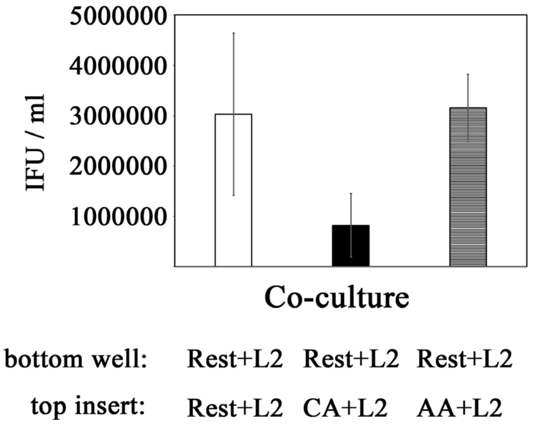

Figure S1: Complex interactions of bystander macrophages on resting mϕ.
Resting mϕ (Rest) were co-cultured with by macrophages of the resting (Rest), classically activated (CA), or alternatively activated (AA) phenotype. IFUs were recovered from the resting mϕ in the bottom well to study the impact of bystander macrophages of the top insert onto the infectious cycle of Chlamydia trachomatis in the bottom well. Recovery of IFU/ml was 3028800 for resting, 820300 for CA, and 3155000 for AA MO. The data are suggesting a positive effect of resting and AA bystander MO, and negative effect of CA MO on targeted resting macrophages.
